# Supplementary material for: Fathers involvement in child feeding and its associated factors among fathers having children aged 6 to 24 months in Antsokia Gemza Woreda, Ethiopia: Cross-sectional study
Source: PLoS One. 2022 Nov 28;17(11):e0276565. doi: 10.1371/journal.pone.0276565 (PMC9704664; doi:10.1371/journal.pone.0276565)
Supplement: S1 File — (DOCX) [file pone.0276565.s001.docx]

DEMOGRAPHIC INFORMATION QUESTIONNAIRE (English version)

1.Age……………….

2. Sex……………….

3.Marital status:..........................

4.Educational status.....................

6. Religion ……………………….

8. Occupational status

9. How many years have you been epileptic? __________

10. Do you have history of admission in health institutions for epilepsy----

11. Where is your Residence rural/urban? -------

12. Number of AEDs you take….

Semi-structured in-depth interview guide (English version)

I have to use this list of questions that will guide us through a conversation about your experience with epilepsy. But we don’t have to stick to them. If we start talking about something you think is important that’s ok. Or, if we run out of things to talk about then I can always use some of these questions to keep the interview going. It doesn’t have to be adhered to completely: instead, the participant’s response will guide the question.

Project Name:------------------------------ Interviewer:---------------------------------------------

Fake Name of Participant (ID):----------------------------- Date:------------------------------------

Start Time:-----------------End Time:------------------Location:-------------------------------------

Interview probes for young people with epilepsy

1. What does to live with epilepsy mean to you?
2. How would you describe your experience of living with epilepsy? In terms of:

Probe: emotional, social, economic, physical and health care service related situations. Can you give me an example of when that happened and how it happened? What did it feel like for you? How would you explain it or make sense of it

1. What situations experienced connected with your life due to having epilepsy
2. When you think about having epilepsy, what emotions do you feel?
3. How do you feel when you have a seizure? What do you do at that time? How do you think your teachers and classmates look at your illness? How do you feel about the teachers’ and peers’ responses

How do you overcome those negative emotions related with your epilepsy condition probe: Emotional control, Emotional expressivity, Control over unpleasant thoughts,

Stress management, Willpower and others

1. Please describe how your illness influences your social life.

Probe: Social contribution, Social acceptance, Social support, Personal relationship Social intimacy Social functioning, Self-esteem, Autonomy, Satisfaction with life roles. Would you tell me about your school life; how do you get on with your teachers, schoolmates and your courses? Please talk about how you get along with your parents and siblings . How do people treat you when they find out you have epilepsy?

1. What coping mechanisms do you use at time of challenges in social life?
2. How do you describe your physical experience related with epilepsy?

Probe: somatic complaints, Mobility, problems with performing activities of daily living

1. Could you tell me about any physical injury you have faced during seizure

Probe: Tongue bite, burn, other body injury and getting wet during seizure

1. How you overcome challenges you faced due to being an epileptic patient(related with physical experience)
2. How do you describe your economic status related with epilepsy condition

Probe: Satisfaction with living conditions (for example, financial situation) your income, debt, job loss, the cost you incur for treatment of epilepsy and opportunity cost

1. What has been your experience with this health facility?
2. What did having epilepsy mean to you before you were diagnosed? Can you tell me about the medical support you received? ; What did it mean to be diagnosed with epilepsy?
3. How do you describe the treatment you received in health facility related your condition?

Probe: utilization AED, psychotherapy, other non-medical support, during admission or OPD, challenges you faced in health facility and your satisfaction with service

1. What do you think can be done for PWE? Would you ever consider changing your medication or your dosage and why is that?
2. How do you overcome challenges you faced in health facility?
3. Is there anything else you would like to share with me? Anything you think I should know about having epilepsy and traditional way of treatment for PWE

Thank participant for participating in the interview.

Observational checklist for care context of epilepsy in Bahidar city government referral hospitals

1. Name the hospital-----
2. The usual prescribed AEDs------------
3. The available drugs at the respected pharmacy of the hospital----------
4. Clinic in where epilepsy cases seen------------------
5. Are health professionals busy to give the service ------------
6. Is it overcrowded----------------
7. Do health professionals respect their clients-------------
8. Do health professionals do special activities such as psychotherapy, counseling and giving adequate information for epileptic young people? -------
