# Supplementary material for: Fathers involvement in child feeding and its associated factors among fathers having children aged 6 to 24 months in Antsokia Gemza Woreda, Ethiopia: Cross-sectional study
Source: PLoS One. 2022 Nov 28;17(11):e0276565. doi: 10.1371/journal.pone.0276565 (PMC9704664; doi:10.1371/journal.pone.0276565)
Supplement: Attachment — Submitted filename: PONE-D-22-14665_R1_reviewer comments.pdf [file pone.0276565.s004.pdf]

PONE-D-22-14665_R1: Fathers Involvement in Child Feeding and Its Associated Factors Among Fathers Having Children Aged 6 to 24 Months in Antsokia Gemza Woreda, Ethiopia: Cross- Sectional Study

Thank you for the opportunity to review the revision of the manuscript. This manuscript is very much improved and it is good to see that the authors have addressed the issues based on reviewers' feedback. However, the validation of the questionnaire issue remains.

Methods:

Please provide clearer illustration of the map (Figure 1).

The key area of concern relates to the validation of the questionnaire. There is insufficient evidence provided to support the claims that the instruments used are reliable and valid.

Detailed description is needed for the development and validation of the questionnaire. "*Literature is reviewed to develop a questionnaire and it was validated*…"

Please provide psychometric properties of the instrument.

Overall comments:

It is strongly suggested to send the paper for proof-reading as there are grammatical and sentence structure errors.
