# Supplementary material for: Fathers involvement in child feeding and its associated factors among fathers having children aged 6 to 24 months in Antsokia Gemza Woreda, Ethiopia: Cross-sectional study
Source: PLoS One. 2022 Nov 28;17(11):e0276565. doi: 10.1371/journal.pone.0276565 (PMC9704664; doi:10.1371/journal.pone.0276565)
Supplement: Attachment — Submitted filename: Reviwer comment_PONE-D-22-14665_R2.pdf [file pone.0276565.s006.pdf]

PONE-D-22-14665_R2

Thank you for the opportunity to review the second revision of the manuscript.

All the comments have been addressed. The paper will make an important contribution to the literature.
