# Supplementary material for: Fathers involvement in child feeding and its associated factors among fathers having children aged 6 to 24 months in Antsokia Gemza Woreda, Ethiopia: Cross-sectional study
Source: PLoS One. 2022 Nov 28;17(11):e0276565. doi: 10.1371/journal.pone.0276565 (PMC9704664; doi:10.1371/journal.pone.0276565)
Supplement: Attachment — Submitted filename: PONE-D-22-14665_reviewer comments.docx [file pone.0276565.s002.docx]

**Fathers Involvement in Child Feeding and Its Associated Factors Among Fathers**

**Having Children Aged 6 to 24 Months in Antsokia Gemza Woreda, Ethiopia: Cross- Sectional Study**

**Background:**

Please provide information to highlight the significance of the study. Explain the father’s role in child feeding and how the father can influence the nutritional outcomes among children. Provide the association or relationship between malnutrition with father involvement in child feeding with literature support.

**Methods:**

My main concern is the questionnaire used for the study. Detailed description is needed for the development and validation of the questionnaire. It is not sufficient with one sentence “*Literature is reviewed to develop a questionnaire (23).*”

The questionnaire is consisted of 4 parts: Father involvement, Knowledge, attitudes. Culture of Fathers about Father Involvement in Child Feeding.

Please provide information on the number of items, the scoring methods and interpretation of the scores for each part of the questionnaire.

Item 3 to 9 for knowledge questions are more related to practices towards father involvement in child feeding as we cannot expect a dichotomous response for these questions and it is not appropriate to assess knowledge, for instance: Item no. 3 – *“fathers involvement in child feeding in providing advice or suggestions on diet”* and item no.6 – *“fathers involvement in child feeding in prepare and cooking a meal for a child.”*

Apart from that, items for father involvement activities in child feeding (*assist mother with household chores*, *accompanying mother for child health clinics,* *allowing other family members/relatives to support the mother after delivery, transporting the child to health clinics, looked after your child when the mothers were not around*). These questions are more related to activities of looking after the child, shar burden of the wife, not measure father involvement in child feeding directly.

Is it possible multiple submissions as the father might have two children range 6 months to 24 months?

Please provide justification of pilot test with 5% of sample size.

Please provide explanation on types of bivariate analysis and multivariate logistic regression analysis.

**Results:**

Please provide justification for 100% response rate.

Suggest to remove figure 1, 2 and 3.

**Discussion:**

The authors have discussed the study results and have attempted to link it with previous studies. However, the result was not been analyzed and argued critically.

Please provide description on “Alive &Thrive's activities in Ethiopia” as background information.

**Overall comments:**

This paper requires proof-reading as there are grammatical and sentence structure errors. Citing and referencing skills can be further improved. There are some claims that are not having references. As a reader, I had difficulties to follow which of the sentences / ideas were from the references and which were authors own ideas. I hope the authors be more precise with the referencing.
