# Supplementary material for: Fathers involvement in child feeding and its associated factors among fathers having children aged 6 to 24 months in Antsokia Gemza Woreda, Ethiopia: Cross-sectional study
Source: PLoS One. 2022 Nov 28;17(11):e0276565. doi: 10.1371/journal.pone.0276565 (PMC9704664; doi:10.1371/journal.pone.0276565)
Supplement: Attachment — Submitted filename: Response to Reviewers.docx [file pone.0276565.s005.docx]

**RESPONSE TO EDITOR AND REVIEWERS**

**RESPONSE TO EDITOR**

**RESPONSE: Thank you for coordinating the review process and fruitful comments. We have revised the manuscript and addressed Reviewer’s comments.**

**Journal Requirements:**

**COMMENT:** Please review your reference list to ensure that it is complete and correct. If you have cited papers that have been retracted, please include the rationale for doing so in the manuscript text, or remove these references and replace them with relevant current references. Any changes to the reference list should be mentioned in the rebuttal letter that accompanies your revised manuscript. If you need to cite a retracted article, indicate the article’s retracted status in the References list and also include a citation and full reference for the retraction notice.

**RESPONSE: We have reviewed our reference list and we have got it complete and correct**

**Additional Editor Comments:**

**COMMENT:** Dear Authors, Thank you for the revised manuscript. However, there are minor issues that need to be addressed as per reviewer comments and the need for proof reading

**RESPONSE: We thank the reviewer for kind words and for fruitful comments. We have revised our paper by correcting** grammatical and sentence structure errors.

**RESPONSE TO REVIEWER 1:**

**Reviewer #1: All comments have been addressed**

**RESPONSE: We thank the reviewer for kind words. We have revised our manuscript based on the comments as described below.**

**REVIEWER COMMENT:** Reviewer #1: It is not clear in Table 6; the income is less than 500 and etc. What is the minimum wage for a family should be? 500 is equivalent to how many dollars (USD)?

**RESPONSE: We thank the reviewer for kind words and comments. We would like to notify for you that we add the term Ethiopian birr to make clarity for the income. Now a days 52.811 Ethiopian birr is equivalent to 1 USD dollars. So that 500 Ethiopian birr is Equivalent to 9.467 USD dollars. In Ethiopia, the household is deemed as living in poverty if the per capita consumption is less than equal Birr 3781, so that minimum wage for a family to classify as poor is less than 500 birrs.**

**REVIEWER COMMENT:** I don't understand in financial disclosure no funders but this work is supported by Bill and Melinda gates as well?

**RESPONSE: We thank the reviewer for kind words and comments. We have not received specific funding starting from proposal development to report writing. We ensured for you that, we have not received specific fund from Bill and Melinda gates.**

**REVIEWER COMMENT:** Besides this study findings will be presented at conference, how is the data will be conveyed to the general public in Ethiopia?

**RESPONSE: We thank the reviewer for kind words and comments. We will have plan to contact Ethiopian Ministry of Health to use our data for policy development and to design intervention based on our finding.**

**RESPONSE TO REVIEWER 2:**

PONE-D-22-14665_R1: Fathers Involvement in Child Feeding and Its Associated Factors Among Fathers Having Children Aged 6 to 24 Months in Antsokia Gemza Woreda, Ethiopia: Cross- Sectional Study 1 Thank you for the opportunity to review the revision of the manuscript. This manuscript is very much improved and it is good to see that the authors have addressed the issues based on reviewers’ feedback. However, the validation of the questionnaire issue remains.

**RESPONSE: We thank the reviewer for kind words. We have revised our manuscript based on the comments as described below.**

**REVIEWER COMMENT:** Please provide clearer illustration of the map (Figure 1).

**RESPONSE: We thank the reviewer for kind words. We have been readjusted it based on your comment**

**REVIEWER COMMENT:** -

Methods: The key area of concern relates to the validation of the questionnaire. There is insufficient evidence provided to support the claims that the instruments used are reliable and valid. Detailed description is needed for the development and validation of the questionnaire. “Literature is reviewed to develop a questionnaire and it was validated…” Please provide psychometric properties of the instrument.

**RESPONSE:** **we adapt the questionnaire with necessary modification from the study conducted in North East Nigeria (27) which aimed to asses fathers’ knowledge, attitude and practices of fathers on their role to support recommended IYCF practices. In that study the questionnaire preliminary draft questionnaire developed by the consultant was reviewed by SC. It was then pre-tested in the field, revised and completed for the field survey, but psychometric analysis was not performed. Furthermore, as we informed you before, we have done pretest since pre-tests also provide the most direct evidence for the validity of the questionnaire; More over to determine the content validity of the questionnaire, the collected views, as well as comments experts in the fields of nutrition, biostatics, and epidemiology, were taken. We thank the reviewer for kind words and such an interesting comment and we will consider your comment as a title for future study which entitled with tool validation on fathers Involvement in Child Feeding and Its Associated Factors by using Confirmatory factor analysis.**

**REVIEWER COMMENT:** Overall comments: It is strongly suggested to send the paper for proof-reading as there are grammatical and sentence structure errors.

**RESPONSE: Thank You for fruitful comments. We have revised our paper by correcting grammatical and sentence structure errors.**
