# Supplementary material for: Fathers involvement in child feeding and its associated factors among fathers having children aged 6 to 24 months in Antsokia Gemza Woreda, Ethiopia: Cross-sectional study
Source: PLoS One. 2022 Nov 28;17(11):e0276565. doi: 10.1371/journal.pone.0276565 (PMC9704664; doi:10.1371/journal.pone.0276565)
Supplement: Attachment — Submitted filename: Response to Reviewers.docx [file pone.0276565.s007.docx]

**RESPONSE TO EDITOR AND REVIEWERS**

**RESPONSE TO EDITOR**

Thank you for submitting your manuscript to PLOS ONE. After careful consideration, we feel that it has merit but does not fully meet PLOS ONE’s publication criteria as it currently stands. Therefore, we invite you to submit a revised version of the manuscript that addresses the points raised during the review process.

**RESPONSE: Thank you for coordinating the review process and fruitful comments. We have revised the manuscript and addressed both editor and Reviewer’s comments.**

**Journal Requirements:**

**COMMENT:** Please review your reference list to ensure that it is complete and correct. If you have cited papers that have been retracted, please include the rationale for doing so in the manuscript text, or remove these references and replace them with relevant current references. Any changes to the reference list should be mentioned in the rebuttal letter that accompanies your revised manuscript. If you need to cite a retracted article, indicate the article’s retracted status in the References list and also include a citation and full reference for the retraction notice.

**RESPONSE: We have reviewed our reference list and we have got it complete and correct**

**Additional Editor Comments:**

**COMMENT:**

Many thanks for the time and effort in revising the manuscript. Overall, most of the comments have been addressed. However, we noted that although Table 6 have been added the term Ethiopian birr to make clarity for the income, it was noted that

1) Table 1 should also have the added term Ethiopian birr to made clear on the household monthly income.

2) To include an NB that a) 52.811 Ethiopian birr is equivalent to 1 USD dollars. b) minimum wage for a family to classify as poor is less than 500 birrs.

**RESPONSE: We thank the editor for kind words and for fruitful comments. We have revised it**

1) **by adding word In Ethiopian birr in table 1 in household monthly income part.**
2) **by adding remember note below table 1 and table 6 as: a) 52.811 Ethiopian birr is equivalent to 1 USD dollars. b) minimum wage for a family to classify as poor is less than 500 birrs.**

**COMMENT:** There is also a need to proof read the manuscript before acceptance.

**RESPONSE: We thank the reviewer for kind words and for fruitful comments. We have revised our paper by correcting grammatical and sentence structure errors.**

**RESPONSE TO REVIEWER 1:**

Reviewer's Responses to Questions

Comments to the Author

1. If the authors have adequately addressed your comments raised in a previous round of review and you feel that this manuscript is now acceptable for publication, you may indicate that here to bypass the “Comments to the Author” section, enter your conflict-of-interest statement in the “Confidential to Editor” section, and submit your "Accept" recommendation.

Reviewer #2: All comments have been addressed

**RESPONSE: We thank the reviewer for kind words.**

2. Is the manuscript technically sound, and do the data support the conclusions?

The manuscript must describe a technically sound piece of scientific research with data that supports the conclusions. Experiments must have been conducted rigorously, with appropriate controls, replication, and sample sizes. The conclusions must be drawn appropriately based on the data presented.

Reviewer #2: Yes

**RESPONSE: We thank the reviewer for kind words.**

3. Has the statistical analysis been performed appropriately and rigorously?

Reviewer #2: Yes

**RESPONSE: We thank the reviewer for kind words.**

4. Have the authors made all data underlying the findings in their manuscript fully available?

The PLOS Data policy requires authors to make all data underlying the findings described in their manuscript fully available without restriction, with rare exception (please refer to the Data Availability Statement in the manuscript PDF file). The data should be provided as part of the manuscript or its supporting information, or deposited to a public repository. For example, in addition to summary statistics, the data points behind means, medians and variance measures should be available. If there are restrictions on publicly sharing data—e.g. participant privacy or use of data from a third party—those must be specified.

Reviewer #2: Yes

**RESPONSE: We thank the reviewer for kind words.**

5. Is the manuscript presented in an intelligible fashion and written in standard English?

PLOS ONE does not copyedit accepted manuscripts, so the language in submitted articles must be clear, correct, and unambiguous. Any typographical or grammatical errors should be corrected at revision, so please note any specific errors here.

Reviewer #2: Yes

**RESPONSE: We thank the reviewer for kind words.**

6. Review Comments to the Author

Please use the space provided to explain your answers to the questions above. You may also include additional comments for the author, including concerns about dual publication, research ethics, or publication ethics. (Please upload your review as an attachment if it exceeds 20,000 characters)

Reviewer #2: Thank you for the opportunity to review the second revision of the manuscript.

All the comments have been addressed. The paper will make an important contribution to the literature.

**RESPONSE: We thank the reviewer for kind words.**

7. PLOS authors have the option to publish the peer review history of their article (what does this mean?). If published, this will include your full peer review and any attached files.

If you choose “no”, your identity will remain anonymous but your review may still be made public.

Do you want your identity to be public for this peer review? For information about this choice, including consent withdrawal, please see our Privacy Policy.

Reviewer #2: No

**RESPONSE: We thank the reviewer for kind words.**
